# Heart Rate Variability Spectral Analysis for Monitoring Autonomic Activation in a Donkey Involved in Animal-Assisted Therapy: A Single Subject Design During Animal-Assisted Therapy Sessions

**DOI:** 10.3390/vetsci12121131

**Published:** 2025-11-28

**Authors:** Michele Panzera, Alessandra Statelli

**Affiliations:** 1 Dipartimento di Scienze Veterinarie, Università degli Studi di Messina, Via Palatucci sn, 98168 Messina, Italy; mpanzera@unime.it; 2 Centro Universitario Specializzato per gli Interventi Assistiti con gli Animali, Università degli Studi di Messina, 98168 Messina, Italy; 3 Dipartimento di Prevenzione Veterinaria Asp Ragusa, Via S. Giovanni Bosco 6, 97100 Ragusa, Italy

**Keywords:** AAS, animal welfare, donkeys, human–animal interaction

## Abstract

*Background*: Only a limited number of studies have investigated objective indicators to assess donkey welfare during Animal-Assisted Services. *Objective*: The present research follows a single-subject design and its objective is to evaluate the neurovegetative indicators of the well-being of a donkey through spectral analysis of the R-R signal in the frequency domain. *Methods:* The experimental protocol of the Animal-Assisted Therapy project involved one donkey, previously selected through behavioral protocol evaluation, and ten patients with a diagnosis of paranoid schizophrenia. Spectral analysis of the R-R signal in the frequency domain was performed, providing objective data on the activity of the sympathetic and parasympathetic nervous systems of the donkey (before, during, and after the sessions). *Results*: The significance of the variations, both statistically significant and not, supports the hypothesis that the affiliative human–donkey interaction within the context of AAS is associated with modifications in the neurovegetative components of the donkey involved in AAT. *Conclusions:* These findings highlight the importance of objective and non-invasive monitoring tools to detect early signs of discomfort in donkeys involved in AAT, supporting the development of selection and management strategies that safeguard animal welfare.

## Simple Summary

This study investigated heart rate variability (HRV) as a non-invasive method to monitor changes in autonomic activation in a donkey involved in Animal-Assisted Services (AAS). By applying a heart rate monitor, we recorded parameters indicating the variations in physiological arousal of the donkey suitable for sessions of Animal-Assisted Therapy, without causing stress or discomfort. The finding suggests that HRV spectral analysis may provide valuable insight into the welfare of donkeys during therapeutic activities, supporting animal well-being and the effectiveness of animal-assisted interventions.

## 1. Introduction

Research on animal emotions and affective processes during Animal-Assisted Service (AAS) is a new focus of animal welfare science, and the correlation of ethological and physiological parameters provides new and significant elements to measure animal behavioral and physiological engagement [1,2,3,4]. Measuring animals’ emotions presents some inherent difficulties [5,6,7]), such as the wide variety of behavioral patterns among species [5].

Recent literature has increasingly explored affective and affiliative processes in equids, particularly in the context of animal-assisted interventions (AAS). Although the interpretation of discrete affective states and autonomic responses in donkeys remains limited, several studies have shown that equids display behavioral and physiological patterns consistent with variations in affective valence and arousal. For example, Mendonça et al. (2019) [8] reported that horses involved in equine-assisted therapy exhibit changes in heart rate variability (HRV) and behavioral indicators that reflect their level of comfort, alertness, or challenge during human–animal interactions. Similarly, Seganfreddo et al. (2023) [4] demonstrated that donkeys show measurable behavioral adjustments and HRV variations when exposed to situations with different emotional relevance, suggesting that non-invasive physiological measures can be informative for assessing affective responses in this species.

More broadly, research on affective states in domestic ungulates supports the feasibility of investigating emotional valence using physiological markers. Ramirez Montes de Oca et al. (2024) [9] identified thermographic asymmetries associated with positive and negative affective conditions in calves, reinforcing the view that animals can exhibit measurable physiological correlates of affective processing. Furthermore, qualitative research in the AAS field emphasizes the importance of considering the animal’s behavioral engagement and social motivation during therapeutic sessions [10], highlighting that affiliative tendencies and social responsiveness contribute to the quality of human–animal interactions. These findings collectively support the relevance of examining changes in arousal and affiliative behavior in donkeys during AAS, without assuming complex emotional intentionality.

Species-specific ethograms, animal proxemics, and the analysis of posture, attitudes, and vocalizations [11,12,13,14] are often used, because they are rapid and harmless; however, no single indicator can provide a complete picture of an animal’s emotional status. Yet, the need for multiple indicators entails the risk of their non-concordance [11]. Another ethological parameter is facial emotional expression, which must be based on reliable, scientifically validated, and non-invasive measurement methodologies [15,16].

Facial expressions are widely studied in humans as a parameter of psychological and emotional experiences. However, they are rarely used in animal studies, with the exception of the emerging field of research concerning postural attitudes and ongoing facies of painful symptomatology in mice [17], rabbits [18], horses [19], cats [20], and donkeys [21,22,23].

In the study of animal emotions, positive emotion states have received less empirical consideration than negative emotions; however, awareness of the importance of positive experiences is increasing, as is the characterization of what constitutes a positive experience for an animal [24,25,26,27,28].

Facial expressions, posture analysis, and vocalizations, as manifestations of an animal’s emotional state, require an objective evidence base; for example, the evaluation of heart rate variability (HRV) can provide evidence for the involvement of the central neurophysiological processes underlying the responses to stress or the different levels of well-being of both farm and companion animals [29,30,31,32].

Heart rate variability (HRV) is an indicator of altered states of autonomic and physiological regulation, which can occur in situations of stress in humans [33] and non-human animals [29,31,32,34,35,36]. HRV assessment has also been used in autonomic nervous system research on animals [34]. In donkeys, it has been studied as an indicator of stress [4], with one study comparing HRV parameters with two different training methods [37] and another with HRV analysis before, during, and after an AAS session [38]; these studies support the possibility of the use of heart rate variability as a reliable indicator of emotional activation and stress response in equids exposed to different types of human interaction and training conditions, particularly when interpreted in relation to concurrent behavioral data, supporting a more integrated evaluation of autonomic and physiological responses.

HRV assessment has also been applied to study the functioning of the autonomic nervous system [39], to analyze changes in the sympatico vagal balance linked to diseases and psychological and environmental stressors [40,41,42,43,44,45,46] or to analyze individual characteristics such as temperament and coping strategies [47,48,49,50,51,52].

On the adaptive side of coping, a higher use of active coping was associated with lower heart rate variability (HRV) in a sample of young healthy men, while there were no significant correlations between HRV and coping for women [53]). In another study, women who reported higher use of problem-focused techniques (i.e., seeking social support) had higher baseline systolic and diastolic blood pressure, as well as higher blood pressure reactivity, during a mental arithmetic task [54].

Recent literature highlights that HRV can be considered a useful physiological indicator associated with affective states in equines and other domestic species. For example, in some studies [4,8] discuss HRV within the framework of affective state in horses and donkeys, and Ramirez Montes de Oca et al. (2024) [9] reports physiological indicators associated with affective valence in calves.

Changes in cardiac activity are considerably influenced by behavior; in particular, those related to motor activity (kinetic, exploratory, grazing, social activity, etc.) [55,56] can be compared with non-motor or psychological/emotional components. Another important aspect is that variations in cardiac activity can be anticipatory, occurring before the expression of any behavior. These anticipatory variations in cardiac activity have been observed in numerous animal species; for example, it is not rare to observe tachycardia several seconds before the emergence of a behavioral change, just as a cardiac response can be maintained beyond the expression of the specific behavioral event with which it was initially related.

These relationships can be considered as specific circulatory demands through which the cardiovascular components of affective-emotional responses are expressed.

For this reason, among the physiological indicators of animal reactivity or emotional stability, through its frequency and variability, the monitoring of cardiac activity constitutes a valid objective evaluation parameter to determine the level of emotional involvement in experimentally induced situations.

Exploring the underlying autonomic control activity of HRV through spectral analysis of the R-R signal in the frequency domain provides objective data reflecting the activities of the sympathetic and parasympathetic nervous systems [57,58,59,60].

Attempts to objectively identify stress from physiological signals have involved temporal [61], spectral [57], and nonlinear [62] measures; nevertheless, conventional algorithms have not been able to obtain a reliable and exact method to discern between stress states and rest.

The generic physiological response to combat stress, called “fight-or-flight,” is largely driven by the sympathetic nervous system (SNS). However, the responses to stress are not exclusively sympathetic; the parasympathetic nervous system (PNS) participates in the response to stress with manifestations such as crying or emptying the bladder.

In humans sympathetic activity tends to increase the heart rate (HR) and decrease the heart rate variability (HRV), while parasympathetic activity tends to decrease the HR and increase the HRV [39]. Through power spectral analysis of the HRV, it is possible to determine the low-frequency (LF) and high-frequency (HF) power of the HRV [41]. The LF power is primarily influenced by sympathetic nervous activity, as well as baroreceptor responses, and a correlation was observed between LF power and muscle sympathetic nerve activity in healthy subjects [58]. The HF power is influenced by respiratory fluctuations, enhanced by vagal stimulation, and attenuated by inhibiting muscarinic receptors or vagal blockades, suggesting that the HF power indicates parasympathetic processes. Hence, power spectral analysis of the HRV provides a means to indirectly investigate autonomic nervous system function, reliably detect the affective states of the subject under study, and provide comfort/discomfort indicators. The heuristic approach to monitoring animal affective state, using the methods and tools of applied veterinary ethology, involves the construction of an evaluation model with known input variables (neurovegetative parameters). Most contemporary approaches for investigating emotions in animals rely on interpreting behavioral patterns together with physiological indicators. Some of these measures—for example, changes linked to physiological stress responses—can reveal whether an animal is experiencing heightened arousal, although they provide limited information about the positive or negative valence of that state [63,64]. Other approaches, such as cognitive bias assessments, can offer insights into whether an animal is in a generally more positive or more negative affective condition [14]. The ethological variables were determined through the comparative semantic study of both the postures and facies of a horse [65], together with our previous investigations into the topic [38,66,67,68]. From the bibliographical review of the effects of assisted therapies with horses and donkeys [69,70,71,72,73,74,75,76,77,78,79,80], it emerged that, in AAS with a horse, the therapy aimed at achieving postural and kinetic physical benefits and, more generally, kinesics, while in AAS with a donkey, the therapy aimed at improving mental health and was used in co-therapeutic approaches for neurodiversity.

In AAS, the assessment of animals’ affiliative tendencies [10,37] is determined via the measurement of their activation state. In various sessions, modifications in the level of arousal [4,8,14]—facilitated by the autonomic nervous system (ANS), the hypothalamic–pituitary axis, and the adrenal gland (HPA)—can be evaluated through adaptive physiological reactions such as the heart rate (HR), blood pressure (BP), breath frequency (BF), pupil diameter, sweat, corticosteroids levels, and neurotransmitters [81]. These neurovegetative responses are analogous to those observed in humans [82]. Through the correlation of ANS physiological profiles and the ethological parameters of experience, it is possible to assess animal emotions. In humans, a meta-analysis of 22 ANS parameters proved that changes in cardiovascular parameters enable differentiation between positive and negative affective-emotional components of emotions [83]. Other studies have shown that 11 ANS parameters (cardiovascular, electrodermal, and respiratory) vary (with a precision of 85%) between fear, sadness, and neutral emotional responses [84].

To date, no published studies have investigated heart rate variability (HRV) or other objective physiological indicators in donkeys involved in animal-assisted services, and no protocols exist for monitoring donkeys during AAT. For this reason, our work aims to provide an initial contribution in two directions:

(1) To support the preliminary selection of suitable subjects from a group of donkeys that are not yet experienced for AAT (*AWIN welfare assessment protocol for donkeys*), particularly with regard to behavioral aspects (Avoidance Test, Novel Object Tests, Unknown Person Test);

(2) To propose an approach based on objective physiological monitoring, which may help identify signs of stress in donkeys during AAT sessions, considering the limited knowledge of their behavioral signals and expressions of discomfort.

We aimed to evaluate the neurovegetative indicators of the well-being of a donkey used for AAS through spectral analysis of the R-R signal (cardiac beat-to-beat interval series) in the frequency domain, which could provide behavioral indicators of comfort/discomfort of the donkey. Ethically, it is crucial to ensure that AASs do not evolve into an advanced type of animal exploitation.

## 2. Materials

The present research follows a single-subject design, since our methodological approach involved repeated measurements and intra-subject comparisons across different therapy sessions. All procedures were conducted in strict accordance with the Italian legal requirements (National Directive n. 26/14–Directive 2010/63/UE) and followed the guidelines for the care of animals in behavioral research as established by the Association for the Study of Animal Behavior (ASAB). The study group comprised 8 healthy Sardinian donkeys: six females and two stallions, aged 6 ± 2.20 years, free housed on a social farm within the premises of a religious organization in Ragusa (Italy), without any experience in AAS. Although the animal had no prior experience with animal-assisted therapy (AAT), it was accustomed to human presence and daily interactions with its caregiver and other strange people. It is also important to clarify that the donkeys considered for selection were not used for zootechnical or productive purposes, but were kept for non-productive aims, in a context that allowed regular contact with people and positive human–animal interactions (social farm).

During the day, the animals were kept in a paddock of approximately 2000 m^2^ and transferred at night to an 800 m^2^ paddock with a collective box measuring 70 m^2^. Their daily rations consisted of a single distribution of hay (5.0 kg/animal/day) and commercial concentrate feed (2.5 kg/animal/day, labeled as containing crude protein 13.00%, ether extract oil 3.20%, crude fiber 13.00%, ashes 10.50%, sodium 0.50%, lysine 0.44%, and methionine 0.21%). The animals had continuous access to grazing on about 2000 m^2^, and water was available ad libitum.

Prior to collecting the ethological data, all animals underwent assessment using the “AWIN welfare assessment protocol for donkeys” [85], administered by the same veterinarian trained specifically in animal welfare. All donkeys participating in this preliminary part of the study received a score of 3 out of 5, exhibited a negative skin tent test, and showed no injuries. The evaluation protocol [38] resulted in the selection of one donkey, a seven-year-old female named Adalgisa, who was deemed suitable based on the behavioral assessments and therefore considered appropriate to be involved in the AAT sessions.

Before the experimental phase, the selected donkey underwent a three-month habituation period with the handler (animal coadjutor) involved in the project. During this stage, the animal was gradually accustomed to being led with a halter; it was already familiar with gentle handling and routine manipulation by the caregiver. Moreover, prior to HRV data collection, the donkey was progressively habituated to wearing the girth belt used to secure the heart rate monitor. This preparatory habituation process likely contributed to reducing potential stress during the experimental sessions.

The experimental protocol of the AAT project included 10 patients (5 men and 5 women), with an average age of 55.44 +/− 6.10, a diagnosis of paranoid schizophrenia (ICD-11 code, diagnostic code 295.8, according to the ICF classifier), and no previous experience of AAS. The AAT project was structured with 7 sessions for each patient on a weekly basis. The 70 sessions were video-recorded, and to ensure the validity and completeness of the spectral analysis of HRV, 38 were analyzed, as the remaining 32 had signal distortions that made spectral analysis unusable. The donkey was easily led during the sessions, using a nylon halter and a braided cotton lead rope approximately 2.5 m by its handler, and it was occasionally allowed to move freely within the working area. The AAT sessions were recorded, each including the animal’s approach, presentation, contact, hand control activity (brush and curry), hand conduct (using a lead wire with a snap hook in the halter), and detachment (grooming at the withers, vocalizations, and removal). Each session lasted 20 min for each patient, including approximately 10 min of hand control activity and approximately 10 min in which a short 20/30 m lap was performed with a lanyard. There were two intervention delivery areas: one of approximately 60 m^2^ with a roof and another of approximately 200 m^2^; the choice of the first or second depended on the current climatic conditions.

## 3. Methods

The heart rate (HR) was measured in real time using Polar S610i and Polar V800S Polar^®^ heart rate monitors, scanning every 5 s. The Polar S610i heart rate monitor was placed on the cardiac auscultation area ichthys. Bluetooth technology was used by the operator, and a polar belt equine chest strap was fitted to the donkey. Data were transferred via infrared or Bluetooth port to a PC running the Polar Horse SW 4.0 or Polar Flow Sync software and then processed and graphed according to preset macros. The entire temporal session of the heart rate monitor data was compiled in a spreadsheet to calculate the descriptive statistics (average, minimum, and maximum bpm per group). The HR was recorded for 10 min before (T_0_), 20 min during (T_1_), and 10 min after (T_2_) the AAT session.

### Spectral Analysis of the HRV Frequency Domain

In the frequency-domain analysis method, a spectrum evaluation is computed for the adjacent R-wave (R-R interval or R-R) of the QRS complex of the electrocardiogram (ECG) signal. Spectral analysis of the variations in the R-R time series, carried out using the Kubios Premium software^®^, allows for determination of the fundamental oscillatory components, i.e., the frequency bands. In the software, the spectrum is estimated using an autoregressive model and can be partitioned into different spectral components by applying spectral factorization [86,87]. Kubios HRV 2.1 supports the file format of Polar HRM (*.hrm). Spectrum assessments are then partitioned into very-low-frequency (VLF), low-frequency (LF), and high-frequency (HF) bands. The usually employed limits for these bands in normal human subjects are as follows: 0–0.04 Hz (VLF) and 0.04–0.15 Hz (LF), representing sympathetic and vagal modulation, with sympathetic predominance, and 0.15–0.4 Hz (HF), which represents vagal modulation. The software calculates the peak frequencies of the VLF, LF, HF, LF/HF power ratio, and total spectral power. We made the spectral analysis even more reliable by subjecting the time-series to the Welch protocol for spectral periodograms (Hamming window).

Although the interpretation of HRV indices is mainly based on human studies, our aim was to apply these established analytical approaches to donkeys in order to provide new preliminary species-specific data.

## 4. Statistics

The experimental heart rate (HR) data obtained were analyzed using the Friedman test for repeated measures and, subsequently, the Wilcoxon test, in order to identify statistically significant comparisons.

## 5. Results

**Figure 1:**
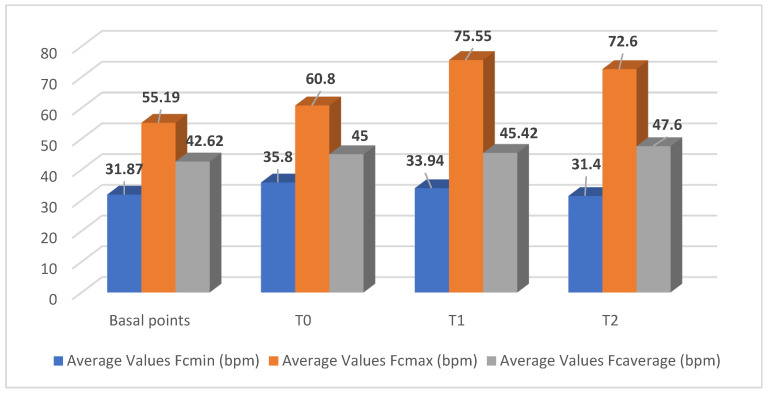
The donkey’s heart parameters in the animal-assisted therapeutic (AAT) session with patients with schizophrenic spectrum disorder.

**Figure 2:**
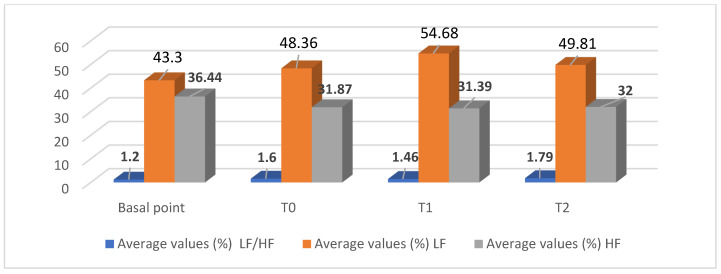
Average values of the spectral analysis of the donkey’s heart parameters in the animal-assisted therapeutic (AAT) session with patients with schizophrenic spectrum disorders.

The following section reports the obtained results in Table 1 and Table 2 and in Figure 1 and Figure 2. The mean basal HR values were equal to 42.62 ± 3.63, in agreement with the literature [38,88,89,90,91,92], with average Fc_min_ values equal to 31.87 ± 3.07 and average Fc_max_ values equal to 55.19 ± 4.53.

We found statistically significant variations in the mean values during the AAT sessions with patients with schizophrenia spectrum disorders (ICD-11): there were higher mean values of the Fc_max_ at T1 equal to 75.55 ± 18.44 and, at T2, which were equal to 72.60 ± 14.70 (*p* < 0.05), as well as the mean values of the mean Fc_max_ at T2, which were equal to 47.60 ± 3.47 (*p* < 0.05).Our previous investigations highlighted such significant variations in the donkey’s HRV during the sessions [35,86]. In terms of the other available literature on the topic, the investigations appear mostly demoscopic [93] or refer to the influence on the salivary cortisol levels of the donkey of the familiarity of contact with normal young people, aged between 18 and 23 [94].

## 6. Discussions

The results relating to the determination of the most important oscillatory components of HRV in the frequency domain represent an original contribution to the determination of the spectral components of the donkey’s R-R time series. The average percentage basal values of the high-frequency component (HF) of the power spectrum reached 36.44 ± 4.72, and those of the low-frequency component (LF) reached 43.30 ± 4.29, with an average value of the LF/HF ratio of 1.20 ± 0.17. The average percentage values of the HRV spectral parameters of the donkey participating in the AAT session showed insignificant increases in the LF/HF ratio and the average percentage value of LF and, conversely, decreases in the average percentage value of HF.

The significance of the set of variations, whether statistically significant or not, of the monitoring indicators of the effects of the AAT session on the temporal trend of HRV, supports the hypothesis that, through the creation of the patient/animal dyad, the care relationship induces variations in the neurovegetative components, reflecting task-related autonomic engagement of the donkey participating in AAT. This suggests the need for adequate rest times between sessions. On the other hand, the percentage increase in the average Fc average values from 42.62 at baseline to 75.55 at time T1 with a +77.26% is consistent with a transient predominance of the sympathetic autonomic component, re-flected by reduced HRV.

The AAT session in this study may represent a context associated with increased au-tonomic activation in the donkey, particularly considering the species high affiliative tendencies. This highlights the importance of appropriate management before and after session, including the identification of activities most effective in supporting physiological recovery and maintaining autonomic balance.

It is usually mandatory to interpret experimental results conditionally, as they can be influenced by the quantity of variables, the operational difficulties, and the complexity of the context. Despite the small sample size (one subject), it is appropriate to highlight, once again, that the path of scientific validation of investigations in the context of the potential therapeutic or educational effects or repercussions on animals of AAS is just beginning and, until non-parametric statistical tools or principal component analyses are common, there will still be a debate as to the necessary sample size and the need for multi-center and randomized studies that researchers apply to the effects of AAS.

The robustness of the experimental data obtained in ethological investigations applied to AAS cannot be derived from the aseptic application of the validation methods of clinical studies. It would be ideal to demonstrate the validity of a research study in the AAS context by attempting to determine how much the results of the study participants represent those of similar individuals outside the study, or by suggesting that its internal validity can be guaranteed by an adequately representative sample population.

It will be interesting to investigate potential correlations between observed asinine facial expressions, as relevant ethological parameters, and the parameters obtained through spectral analysis in the frequency do-main. Correlating behavioral indicators potentially associated with the corresponding values of the LF/HF ratio could provide a valid tool for welfare-oriented ethological moni-toring un the field.

## 7. Considerations

The founding value of Animal-Assisted Services (AAS) lies in the evident and recognized benefit that the human participant (user/patient) derives from the care or educational relationship (dyad human and animal). However, the growing enthusiasm for these integrative therapeutic approaches should not compromise the coherence of the methodology, nor overlook the affective of the animal’s physiological and behavioral state of the other partner—the four-legged one.

The ethics of the interspecific relationship require balancing the costs and benefits for both protagonists of the dyad, even when supported, directed, or guided by a human intervention representative or the animal’s handler.

Donkeys are often selected for interventions without standardized behavioral criteria, and inexperienced animals may show subtle signs of discomfort that are easily overlooked due to the scarce knowledge of the species’ ethological, postural, and expressive repertoire compared with horses or dogs. For this reason, inadequate selection procedures or poorly structured AAT sessions may expose donkeys to stress that is not immediately identifiable through observation alone.

We ensure the protection of the rights of animals involved in AAS only when we verify that their level of affiliative engagement remains ethically acceptable, and we provide them with adequate opportunities for rest and refreshment. The ethical threshold not to be crossed is precisely defined by the scientific evidence that enables us to design Animal-Assisted Therapy and Animal-Assisted Education sessions with prudence, considering the animal’s arousal level. Ignoring these aspects would result in a subtle yet sophisticated form of animal exploitation. Scientific evidence defines the ethical threshold we must respect when designing Animal-Assisted Therapy and Animal-Assisted Education sessions. This evidence guides us to act with prudence and to consider the animal’s arousal level at every stage. Failing to take these aspects into account risks turning the intervention into a subtle yet sophisticated form of animal exploitation.

## Figures and Tables

**Table 1:** Monitoring of the average values of the heart rate parameters of the donkey involved for the AAT session with patients with schizophrenic spectrum disorders.

Time Points	Average Values Fc_min_ (bpm) M ± D.S.	Average Values Fc_max_ (bpm) M ± D.S.	Average Values Fc_average_ (bpm) M ± D.S.
Basal	31.87 ± 3.07	55.19 ± 4.53	42.62 ± 3.63
T_0_	35.80 ± 2.15	60.80 ± 5.85	45.00 ± 2.26
T_1_	33.94 ± 4.15	75.55 ± 18.44 **	45.42 ± 4.83
T_2_	31.40 ± 6.70	72.60 ± 14.70 **	47.60 ± 3.47 **

** *p* < 0.05 vs. basal values.

**Table 2:** Monitoring of the average values of the spectral analysis of heart rate parameters of the donkey involved for the AAT session with patients with schizophrenic spectrum disorders.

Time Points	Average Values (%) LF/HF M ± D.S.	Average Values (%) LF M ± D.S.	Average Values (%) HF M ± D.S.
Basal point	1.20 ± 0.17	43.30 ± 4.29	36.44 ± 4.72
T_0_	1.60 ± 0.59	48.36 ± 6.84	31.87 ± 5.66
T_1_	1.46 ± 0.79	54.68 ± 11.25	31.39 ± 12.62
T_2_	1.79 ± 0.80	49.81 ± 5.59	32.00 ± 11.36

## Data Availability

The data presented in this study are available on request from the corresponding author. The dataset is not public available because it is part of an ongoing research program, and additional analyses are planned for future publications.
